# Evolution and Trends of the Exploration–Exploitation Balance in Bio-Inspired Optimization Algorithms: A Bibliometric Analysis of Metaheuristics

**DOI:** 10.3390/biomimetics10080517

**Published:** 2025-08-07

**Authors:** Yoslandy Lazo, Broderick Crawford, Felipe Cisternas-Caneo, José Barrera-Garcia, Ricardo Soto, Giovanni Giachetti

**Affiliations:** 1 Escuela de Ingeniería Informática, Pontificia Universidad Católica de Valparaíso, Avenida Brasil 2241, Valparaíso 2362807, Chile; yoslandy.lazo@pucv.cl (Y.L.); felipe.cisternas.c@mail.pucv.cl (F.C.-C.); jose.barrera@pucv.cl (J.B.-G.); ricardo.soto@pucv.cl (R.S.); 2 Escuela de Negocios y Economía, Pontificia Universidad Católica de Valparaíso, Amunátegui 1838, Viña del Mar 2580129, Chile; 3 Facultad de Ingeniería, Universidad Andres Bello, Antonio Varas 880, Providencia, Santiago 7591538, Chile; giovanni.giachetti@unab.cl

**Keywords:** bio-inspired optimization, exploration–exploitation balance, bibliometric analysis

## Abstract

The balance between exploration and exploitation is a fundamental element in the design and performance of bio-inspired optimization algorithms. However, to date, its conceptual evolution and its treatment in the scientific literature have not been systematically characterized from a bibliometric approach. This study performs an exhaustive analysis of the scientific production on the balance between exploration and exploitation using records extracted from the Web of Science (WoS) database. The processing and analysis of the data were carried out through the combined use of Bibliometrix (R package) and VOSviewer, tools that made it possible to quantify productivity, map collaborative networks, and visualize emerging thematic trends. The results show a sustained growth in the volume of publications over the last decade, as well as the consolidation of academic collaboration networks and the emergence of new thematic lines in the field. In particular, metaheuristic algorithms have demonstrated a significant and growing impact, constituting a fundamental pillar in the advancement and methodological diversification of the exploration–exploitation balance. This work provides a quantitative framework and a structured view of the evolution of research, identifies the main actors and trends, and raises opportunities for future lines of research in the field of optimization using metaheuristics, the most prominent instantiation of bio-inspired optimization algorithms.

## 1. Introduction

Metaheuristics are advanced search algorithms that apply specific rules to explore and exploit solutions in complex search spaces, being widely used to solve optimization problems in various areas [1,2]. A crucial aspect for the good performance of these methods is the balance between exploration and exploitation. Exploration allows the discovery of diverse solutions in different regions of the search space, facilitating the localization of promising areas. On the other hand, exploitation intensifies the search in these areas to improve existing solutions and accelerate convergence [2,3,4,5,6]. Studying this balance is fundamental, as excessive exploration can slow down convergence, while predominant exploitation can lead to local optima, affecting the efficiency of the algorithm [1,2]. The importance of this balance is widely recognized in the literature, being the subject of analysis in multiple studies that seek to understand and improve the performance of metaheuristics [1,3,7,8].

However, although the importance of exploration–exploitation balancing is widely known, understanding its conceptual and practical evolution in the scientific literature represents a considerable challenge [1,2,6,9,10,11]. Despite its relevance, the literature offers few metrics or accepted methods to measure it in a clear and reproducible way in metaheuristic algorithms [12], which makes its comparative analysis and systematic evaluation difficult. While there are bibliometric studies that provide a comprehensive analysis of the general applications of metaheuristics [13], their approach does not specifically address the exploration–exploitation balance. Currently, to the best of our knowledge, no analysis based on bibliometric metrics has been carried out that would allow us to objectively observe this evolution. Bibliometric analyses are essential to provide a quantitative and structured perspective of the development and trends in a field of study, facilitating the identification of patterns, influences and gaps in research that can guide future studies [14,15]. The application of this type of analysis to the field of study of the balance between exploration and exploitation will make it possible to trace the evolution and publication trends in the field of metaheuristics and to demonstrate the interest of the scientific community in addressing this issue.

In particular, metaheuristics, understood as a prominent subset within the broader field of bio-inspired optimization algorithms, have been consistently recognized for their effectiveness in solving complex optimization problems using adaptive search strategies. According to the taxonomy proposed by Jakšić et al. [16], biologically inspired metaheuristics represent one of the most prominent and prolific categories within bio-inspired methods, owing to their versatility and their capacity to model and regulate the balance between exploration and exploitation in highly nonlinear search spaces.

In this context, the present study aims to quantify and analyze the scientific production related to the balance between exploration and exploitation in metaheuristic algorithms, using as a data source the records indexed in the Web of Science (WoS) database. It is hypothesized that during the last decade there has been a significant increase in the volume of scientific publications that explicitly address the balance between exploration and exploitation in metaheuristics, reflecting a growing interest of the research community in improving the efficiency of these algorithms. It is also expected to identify the main sources of publication, the most influential authors, the collaboration networks and the emerging lines of research associated with this topic, providing a comprehensive view of the development of the area.

Data processing and analysis were performed by the combined use of two specialized tools: Bibliometrix, an R package developed to perform quantitative bibliometric analysis and visualization of results in a reproducible manner [17,18], and VOSviewer, a widely used software for the construction and visualization of scientific network maps, co-authorship networks, co-citation and co-occurrence of terms [19,20]. While VOSviewer excels in the graphical representation of networks and thematic relationships, Bibliometrix provides detailed statistical analysis and high flexibility in data manipulation, thus enabling a more comprehensive and in-depth bibliometric analysis [18,21].

This article proceeds as follows. Section 2 describes the methodology used for the development of the bibliometric analysis, detailing the design of the study, the search strategy in the Web of Science database, and the tools used for data processing and analysis. Section 3 presents the main results, including the evolution of scientific production, the most relevant sources and authors, collaboration networks and emerging thematic trends. Section 4 discusses the most relevant findings, contextualizing their importance in the field of metaheuristics. Finally, Section 5 presents the general conclusions of the study and proposes future lines of research.

## 2. Methods and Data

### 2.1. Study Design

The study employs a quantitative framework, utilizing bibliometric methods to explore the exploration–exploitation balance in metaheuristics. This approach was selected to map and visualize trends in the literature, as well as to evaluate the productivity and impact of research addressing this balance in optimization problems solved through metaheuristic techniques.

To conduct the study, bibliometric techniques are employed, grouped into two complementary categories: scientific performance analysis and scientific mapping. The scientific performance analysis, carried out using the Bibliometrix software (version 4.3.2), includes quantitative metrics such as annual production, author productivity, and document citation, allowing for the measurement of research evolution and relevance. On the other hand, the construction of scientific maps is carried out using VOSviewer (1.6.20) to perform a keyword co-occurrence analysis (identification of thematic cores), assess their relative impact in the scientific literature through overlay visualization mapping of the most frequent author keywords, and analyze co-authorship networks (mapping academic collaborations). On the other hand, the scientific contribution map by country, developed in R using specialized packages, is presented at the end to highlight the geographical distribution of intellectual production. This final component, positioned as the conclusion of the spatial analysis, provides a regional perspective on influence within the field, complementing thematic and collaborative dimensions with a territorial viewpoint. Additionally, the most relevant journals in the topic are identified, emphasizing those that publish most frequently on the balance between exploration and exploitation in metaheuristics. These tools, supported by specialized software (VOSviewer and R), not only enable the identification of patterns in the literature but also provide insights into how strategies addressing the exploration–exploitation balance in metaheuristics are conceptually framed, as well as the academic communities leading their development and the associated international collaboration patterns tied to research geography. The combination of both approaches ensures a comprehensive evaluation, merging quantifiable data with contextual interpretations of the study topic.

### 2.2. Data Source and Search Strategy

The Web of Science (WoS) database, managed by Clarivate Analytics, is a multidisciplinary repository that indexes high-impact scientific publications supported by rigorous peer-review processes. Its extensive coverage includes leading journals in fields such as engineering, computer science, and applied mathematics, which are central to the study of metaheuristics. WoS offers advanced search capabilities and citation metrics, essential for bibliometric analyses. Its selection is justified by its ability to access specialized literature and its flexible interface, which allows the use of Boolean operators and specific filters, ensuring precise and reproducible results.

To optimize the retrieval of relevant literature, a complex Boolean search string was designed, combining logical and proximity operators:

(TS = (“metaheurist*” OR “meta-heurist*”) AND TS = (“balanc*” NEAR/2 “exploration” NEAR/1 “exploitation”)) AND (LA = (“ENGLISH”) NOT (PY = (“2025”) OR DT = (“EARLY ACCESS” OR “BOOK CHAPTER”)))

The TS (Topic Search) operator was used to search for terms in the title, abstract, and keyword fields, maximizing the retrieval of relevant documents. The first clause, TS = (“metaheurist*” OR “meta-heurist*”), covers variations in the central term (e.g., “metaheuristic” or “meta-heuristic”), ensuring comprehensive conceptual coverage. The second clause, TS = (“balanc*” NEAR/2 “exploration” NEAR/1 “exploitation”), uses proximity operators to locate explicit discussions on the exploration–exploitation balance. The truncation in “balanc*” includes related terms (such as “balance” or “balancing”), while NEAR/2 and NEAR/1 define the maximum distance between words, prioritizing contexts where these concepts directly interact in metaheuristic algorithms. Additionally, the search was limited to English-language publications, and documents from the year 2025 (due to the rigorous indexing process of WoS, which can be time-consuming, there may be delays in the availability of recent data [15]), as well as those classified as Early Access or Book Chapter, were excluded. This ensures that only documents classified as Article, Proceeding Paper, and Review Article are included, reflecting the most advanced and peer-reviewed research on the topic.

The dataset analysed in this study was downloaded from Web of Science (WoS) on 16 March 2025. After applying the relevant filters and restrictions, the preliminary number of records retrieved was 880. Following the removal of incomplete records and an additional review, a total of 861 records were included in the analysis.

### 2.3. Analysis Techniques

To examine the scientific production related to the exploration–exploitation balance in metaheuristics, bibliometric techniques were applied, organized into two complementary approaches: scientific performance analysis and scientific mapping. These methodologies allow for a quantitative evaluation of the research evolution in the field and a visual representation of the conceptual and collaborative relationships within the research topic.

Scientific performance was analyzed using various bibliometric indicators to quantify the production and impact of research on the topic. The following metrics were used:

1. Annual scientific production: The number of documents published per year was quantified to identify temporal trends in interest in the topic.
2. Most relevant journals: The primary sources of publication were identified using Bradford’s Law, which classifies publications into productivity zones (core, intermediate, and peripheral), highlighting journals that concentrate the most significant contributions to the research topic [22,23].
3. Productivity and impact analysis of the most relevant authors: Various indicators were analyzed to evaluate the contributions of the most influential researchers in the field, including:
  - Authors’ Production over Time: Tracks the publication and impact trends of the most relevant authors in the research topic.
  - Number of published documents: Total number of articles per author, identifying the most productive authors in the research topic.
  - Total citations: Serves as an indicator of the academic influence of the most relevant authors.
  - h-index: Measures productivity and impact simultaneously, defined as the maximum value h where an author has *h* publications with at least *h* citations each [15].
  - e-index: An indicator that complements the h-index by quantifying the surplus citations not considered by the latter. It is defined as $e=\sqrt{d-h^{2}}$, where *d* represents the total number of citations received by the articles within the h-core, which is the set of the researcher’s *h* articles with at least *h* citations each. This way, the e-index provides a more comprehensive view of scientific productivity and impact [24].
4. Weighted Authorship Credit Allocation: Since raw productivity does not always accurately reflect individual contributions in multi-author publications, a complementary analysis based on weighted credit allocation was incorporated. For each multi-author article, the contribution of the i-th author was estimated using four established metrics from advanced bibliometrics [25,26]:
  - Fractional credit $\left(\frac{1}{n}\right)$ where *n* is the total number of authors;
  - Harmonic credit $\left(\frac{1/i}{\sum_{j=1}^{n}1/j}\right)$, which penalizes co-authors less heavily in later positions;
  - Geometric credit $\left(\frac{2^{n-i}}{\sum_{j=1}^{n}2^{n-j}}\right)$, which assigns greater weight to the early-listed authors; and
  - Arithmetic credit $\left(\frac{n-i+1}{\sum_{j=1}^{n}\left(n-j+1\right)}\right)$, which linearly weights the author’s position.
  Based on these calculations, ranked lists of the top 20 authors were generated for each metric. Finally, researchers who consistently ranked among the top positions across all considered schemes were identified. This approach enabled a more refined identification of the most influential actors within the thematic domain, simultaneously incorporating both productivity and co-authorship structure.
5. Most cited documents: The articles with the highest number of citations within the dataset were identified to recognize the most influential studies in the literature on the exploration–exploitation balance in metaheuristics.

In addition to the quantitative analysis of scientific performance, network analysis techniques were implemented to visualize conceptual and collaborative relationships in the literature. The following approaches were used:

1. Co-occurrence Network: To visualize the thematic structure of the literature on the exploration–exploitation balance in metaheuristics, a keyword co-occurrence map was constructed using VOSviewer (version 1.6.20). This analysis was based on the following methodological criteria:
  Term Normalization: A specialized thesaurus list was applied to group synonymous or conceptually equivalent terms, reducing semantic redundancy and ensuring interpretative coherence of the clusters. For example:
  - Lexical variants (e.g., “meta-heuristic” → “metaheuristics”) and acronyms (e.g., “PSO” → “particle swarm optimization”).
  - Specific terms under general concepts (e.g., “diversity preservation” and “population diversity” → “diversity”).
  Revelance Threholds: To ensure statistical representativeness and network stability, only keywords with a minimum of 18 occurrences in the dataset were included. This threshold was established after analyzing the frequency distribution, discarding marginal terms that could introduce noise or artificially fragment the thematic clusters.
  Association measure: The strength of association was calculated using VOSviewer’s default index (association strength), with a minimum threshold of 2.0 to exclude weak links that could compromise cluster interpretability.
  Additionally, an overlay visualization was generated based on the previously described co-occurrence network. This representation was created using VOSviewer software and allows for the identification of the relative impact of each term within the semantic network by incorporating the metric of average citations per keyword.
  Each node in the map retains its original thematic grouping (co-occurrence cluster), but its coloration varies according to the average citation value of the documents in which the corresponding term appears. Higher values are represented using a chromatic gradient ranging from blue (low impact) to yellow (high impact), thereby providing an additional layer of analysis that enables inferences not only about the thematic structure but also about the bibliometric weight of each component within the studied domain.
2. Collaboration Network: The structure of academic collaboration was analyzed by constructing a co-authorship network, where nodes represent authors and links reflect collaborations between them. In this analysis, only authors who have published at least 5 articles and collaborated with at least one other author were included. This restriction focused the study on prominent researchers with a consolidated trajectory, ensuring the identification of relevant research communities and providing a robust analysis of collaborative interactions in the research topic.
3. Scientific Contribution by Countries: The quantification of the production of published documents by countries was carried out through the integration of the Bibliometrix software and spatial visualization tools in R. The methodology was structured into three interconnected stages:
  - Data Extraction and Normalization:
    The bibliographic database was processed in Bibliometrix (version 4.3.2), extracting the Country field from the institutional affiliations of the authors. To ensure accuracy in territorial attribution, a standardization protocol was implemented to unify lexical variants and acronyms (e.g., “PR China” → “China”, “UK” → “United Kingdom”).
  - Categorization by Productivity Ranges:
    Leading Countries: Unique and contrasting shades were assigned to each of the top five countries with the highest production, prioritizing intense colors for immediate identification (red, purple, orange, dark green, and blue).
    Secondary Ranges: The remaining countries were classified into four intervals based on their total production:
    (a) 61–100 documents: Bright pink
    (b) 20–60 documents: Turquoise
    (c) 6–19 documents: Light green
    (d) 1–5 documents: Light olive green
    (e) No Contribution: Territories without published documents were represented in white.
  - Technical Implementation:
    The vector geometry of the map was linked to the production matrix quantifying published documents using ISO3 country codes, ensuring precise correspondence between geographic entities and bibliometric data.
    The cartographic visualization was generated in R using the ggplot2 and rnaturalearth packages, constructing a georeferenced layer with national polygons adjusted to updated geopolitical boundaries (2023).

This dual approach (Bibliometrix for metrics + R for geo-visualization) enabled the articulation of a rigorous spatial analysis, based on replicable standards and open-source tools.

## 3. Results

### 3.1. Scientific Production in Research on the Balance Between Exploration and Exploitation in Metaheuristics

The bibliometric analysis conducted on the dataset covered a temporal period from 2005 to 2024, enabling a longitudinal evaluation of scientific production in the research topic under study. A total of 861 documents were identified, distributed across 293 journals, suggesting a diversification of publication sources within the research field (Table 1).

A significant volume of 2267 keywords used by the authors was observed, reflecting a broad thematic scope and conceptual diversity in the studies addressed. The average document age was 3.94 years, indicating that the analyzed literature is relatively recent and that the field of study maintains a high dynamic of knowledge updating and renewal. Additionally, the average of 32.08 citations per document demonstrates considerable academic impact within the area.

Regarding authorship structure, a total of 2392 authors contributed to the production of the analyzed documents. A limited number of 40 documents were authored individually, highlighting a predominant trend toward scientific collaboration. Consistent with this trend, the average number of co-authors per document was calculated to be 3.81, suggesting the existence of active collaboration networks and production based on interinstitutional and international synergies.

### 3.2. Annual Scientific Production

**Figure 1:**
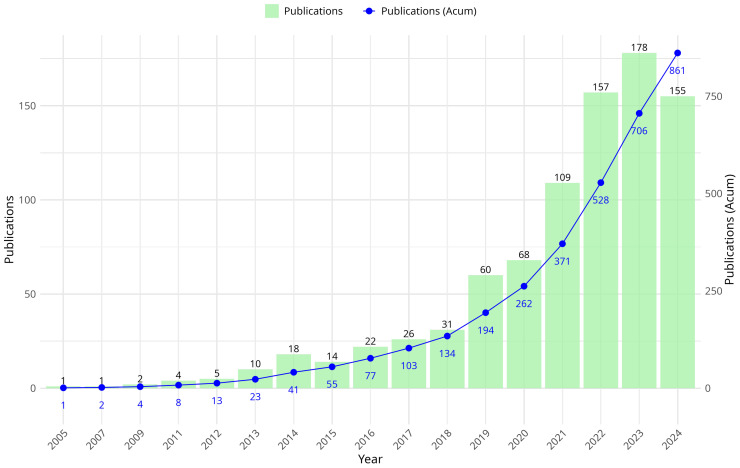
Annual Scientific Production.

Figure 1 illustrates the evolution of scientific production related to the exploration–exploitation trade-off in metaheuristics during the period 2005–2024. The graph features two scales on the vertical axis: the first, on the left, indicates the number of documents published per year, while the second, on the right, represents the cumulative number of publications over time.

During the initial years of the analyzed period (2005–2012), the number of publications was low, with values below 20 documents per year. Starting in 2013, a progressive increase in scientific production is observed, with the cumulative number of documents showing an upward trend and exponential growth behavior.

Between 2018 and 2024, the growth in the number of publications is notably accelerated. In 2019, the total number of documents published on the research topic reached 194, while in 2021 this figure increased to 371. In 2023, the highest value was achieved with 178 publications in a single year.

These data reflect a continuous increase in the number of studies on the exploration–exploitation trade-off in metaheuristics, with higher scientific production in the later years of the analysed period.

### 3.3. Main Publication Sources

**Figure 2:**
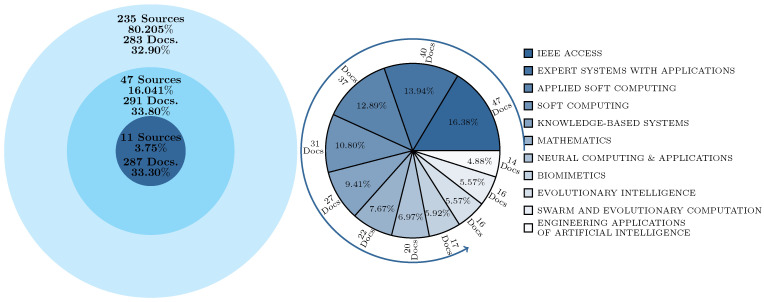
Bradford’s Law.

Figure 2 presents the analysis of the main publication sources using Bradford’s Law, which segments journals into three productivity zones: core, intermediate, and periphery. This classification allows for the identification of the sources that concentrate the highest number of publications on the exploration–exploitation trade-off in metaheuristics.

The first zone, referred to as the core, consists of 11 sources, representing 3.75% of the total journals included in the dataset. In this zone, 287 documents were published, accounting for 33.30% of the total publications. The second zone, termed the intermediate zone, comprises 47 sources, representing 16.041% of the total analyzed sources. In this zone, 291 documents were published, equivalent to 33.80% of the total collected documents. Finally, the third zone, known as the periphery, includes 235 sources, representing 80.205% of the total analyzed journals. In this zone, 283 documents were published, accounting for 32.90% of the total analyzed documents.

The core zone consists of the journals with the highest publication volume on the study topic. Within this category, IEEE Access emerges as the most productive source with 47 documents, followed by Expert Systems with Applications (40 documents), Applied Soft Computing (37 documents), Soft Computing (31 documents), and Knowledge-Based Systems (27 documents). Other notable journals in this zone include Mathematics (22 documents), Neural Computing & Applications (20 documents), Biomimetics (17 documents), Evolutionary Intelligence (16 documents), Swarm and Evolutionary Computation (16 documents), and Engineering Applications of Artificial Intelligence (14 documents). The concentration of publications in these sources underscores their pivotal role in advancing knowledge about the balance between exploration and exploitation in metaheuristics, establishing them as key references in the research field.

### 3.4. Analysis of Productivity and Impact of the Most Relevant Authors

**Figure 3:**
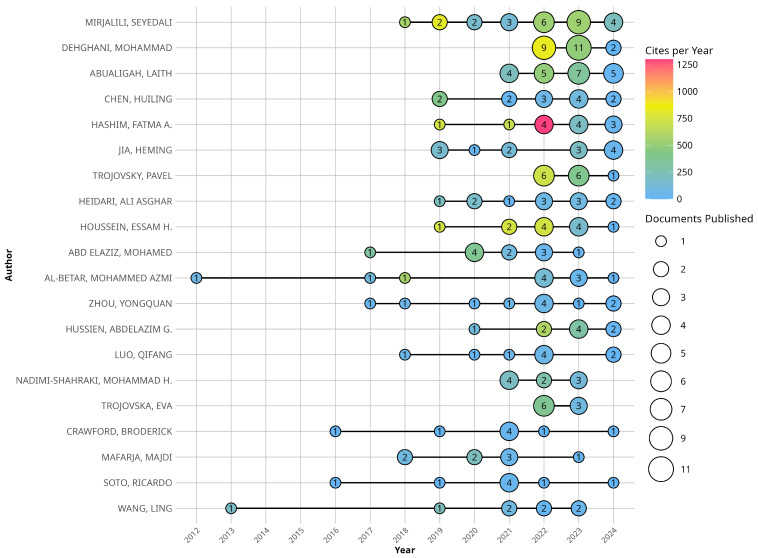
Authors’ Production and Its Impact Over Time.

Figure 3 presents the productivity and impact of the top 20 authors with the highest number of published documents in the analyzed dataset. The documents published by this group of authors span the period from 2012 to 2024. The graph uses a visual representation with circles proportional to the number of documents published by each author in a given year. Additionally, a chromatic scale is incorporated to indicate the impact of each author based on the number of citations received annually.

The analysis of the temporal distribution of scientific production reveals that some authors began contributing to the field more recently, while others have maintained a more extensive publication trajectory. For instance, authors such as Mirjalili, Seyedali, and Dehghani, Mohammad exhibit a sustained high level of productivity in recent years, with significant peaks in the number of publications.

The numerical values within the circles allow for precise quantification of each author’s production in a specific year. It is observed that some researchers show considerable increases in their output starting in 2018, suggesting a growing interest in the topic.

Regarding impact, measured by the number of citations received, the color scale allows for the identification of differences in the influence of each author. Certain years stand out in which some researchers received a higher number of citations, indicating the relevance of their contributions during specific periods.

Table 2 presents the main bibliometric indicators of the 20 most productive authors in the literature on the exploration–exploitation balance in metaheuristics, based on the analyzed dataset. It reports each author’s number of published documents (Docs), total citations according to Web of Science (Cites), and the bibliometric indices h-index and e-index, which assess the impact and relevance of academic contributions.

The most prolific author in the dataset is Seyedali Mirjalili, with 27 documents, followed by Mohammad Dehghani (22) and Laith Abualigah (21). In terms of impact, Mirjalili also leads with 2966 citations, followed by Fatma A. Hashim (2952) and Essam H. Houssein (2385).

The h-index, which simultaneously measures productivity and impact, reaches its highest values for Mirjalili (h = 21), Dehghani (h = 18), and Abualigah (h = 14). Meanwhile, the e-index, which complements the h-index by capturing excess citations, reveals that Fatma A. Hashim has the highest e-index (53) despite publishing only 13 documents. Similarly, Essam H. Houssein achieves an e-index of 48 with 12 documents, suggesting that while his productivity is lower, his work has garnered significant citation impact.

Overall, the data show a concentration of academic influence among a small group of authors, with notable disparities between citation counts and publication volume. This underscores the importance of evaluating not just the quantity of publications, but also their scientific impact.

With the aim of refining the identification of the most influential authors in research on the exploration–exploitation balance in metaheuristics, four weighted authorship credit allocation metrics were applied: fractional, harmonic, geometric, and arithmetic. These metrics allow for the estimation of individual contributions in multi-author articles by taking into account both the total number of authors and the position occupied by each contributor. Table 3 shows the results obtained for the authors who consistently ranked among the top twenty in all the weighted credit allocation schemes evaluated. For each metric, the absolute value of credit calculated is reported and, in parentheses, the position occupied in the corresponding ranking. This procedure complements the analysis based on gross productivity, providing a more precise view of the distribution of author credit in the field studied.

### 3.5. Most-Cited Documents

Table 4 lists the 10 most-cited documents within the analyzed dataset, including details on the first author, publication journal, publication year, and total citations.

The most-cited document is by Fatma A. H. (2019) [10], published in Future Generation Computer Systems, with 92 citations. This is followed by two studies by the same author: one in Applied Intelligence (2021; 67 citations [27]) and another in Mathematics and Computers in Simulation (2022; 58 citations [28]). In fourth place, the work of Malik B. (2022) [29] in Knowledge-Based Systems has accumulated 49 citations, while Mohamed A. E. (2017)’s paper [30] in Expert Systems with Applications ranks fifth with 39 citations.

The analysis reveals that Fatma A. H. dominates the ranking, with four articles among the top 10 most-cited—all published within the last five years in high-impact journals such as Future Generation Computer Systems, Applied Intelligence, Mathematics and Computers in Simulation, and Knowledge-Based Systems [31]. Other authors with notable contributions include Ayyarao T.S.L.V (2022) [6] in IEEE Access with 27 citations, Morales-Castañeda B (2020) [1] in Swarm and Evolutionary Computation with 25 citations, and Trojovský P (2022) [32] in Sensors with 24 citations, while the article by Aydilek I.B. (2018) [33] in Applied Soft Computing concludes the list with 20 citations.

### 3.6. Keyword Co-Occurrence Analysis

**Figure 4:**
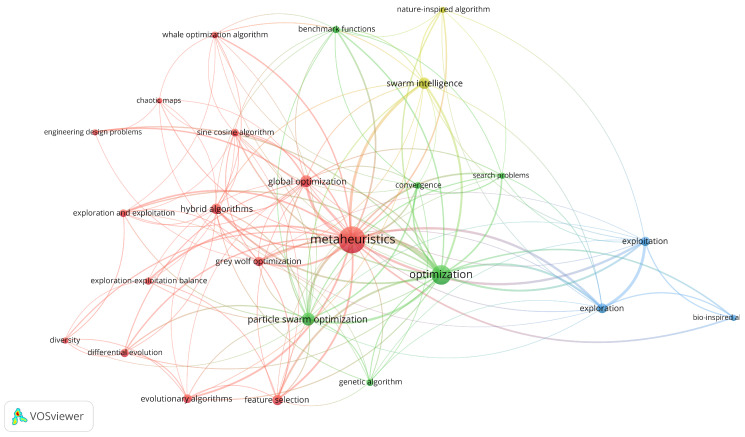
Co-occurrence Network.

To analyze the thematic structure and relative impact of the scientific literature on the exploration–exploitation balance in metaheuristics, a keyword co-occurrence analysis was conducted using author keywords in VOSviewer (version 1.6.20). The analysis was performed on the refined dataset with minimum occurrence (≥18) and association strength (≥2.0) thresholds, including term normalization processes to ensure semantic consistency. The resulting visualization (Figure 4), composed of 25 nodes distributed across four clusters, reflects the semantic and conceptual grouping of terms based on their co-occurrence in the analyzed literature. Each node represents a keyword, and its size corresponds to the term’s frequency of occurrence. The links between nodes indicate how often two terms appear together in the same documents, reflecting thematic cohesion, while the thickness of the links represents the strength of association between terms.

In relation to the interpretation of the thematic clusters derived from the co-occurrence analysis, it should be noted that, in bibliometric analyses, the dispersion of conceptually close terms among different clusters is an expected feature, especially in domains where the conceptual boundaries are fuzzy. This dynamic reflects the semantic complexity of the field rather than a technical flaw of the method. Therefore, clusters should be understood as statistical representations constructed from patterns of co-occurrence or semantic similarity, useful for the exploration of trends, but not as closed thematic categories or definitive divisions of knowledge [34,35,36,37].

The four identified clusters are distributed as follows:

- Cluster 1 (Red): Represents the thematic core of the network and consists of terms referring to specific metaheuristic algorithms and their application domains. It includes keywords such as metaheuristics (the central node of the network), grey wolf optimization, hybrid algorithms, global optimization, evolutionary algorithms, differential evolution, and sine cosine algorithm. Additionally, it features terms like chaotic maps (a technique used in solution diversification within metaheuristic strategies), feature selection (important in optimization methodologies for variable selection in high-dimensional problems), and engineering design problems (reflecting applicability in engineering design). Furthermore, key terms related to adaptive optimization strategies are present, such as diversity, exploration and exploitation, and exploration–exploitation balance. The inclusion of these nodes suggests the relevance of exploration–exploitation dynamics in metaheuristic processes, emphasizing their role in improving algorithm stability.
- Cluster 2 (Green): Focuses on fundamental concepts in optimization processes and computational performance, including nodes such as optimization, genetic algorithm, convergence, particle swarm optimization, and benchmark functions. Its central position in the graph and connectivity with all other clusters indicate that these concepts function as cross-cutting methodological pivots, linking algorithmic techniques with evaluation and testing approaches. This cluster reflects a focus on performance improvement and comparative bio-inspired optimization algorithms.
- Cluster 3 (Blue): Specializes in the relationship between exploration and exploitation, with key nodes such as exploration, exploitation, and bio-inspired algorithms, suggesting an emphasis on modeling balance strategies within bio-inspired metaheuristics. Despite its thematic relevance, its low internal density and peripheral location in the network indicate that while these concepts are fundamental for the theoretical analysis of algorithm behavior, they have not been widely used as author keywords, limiting their visibility in the bibliometric analysis.
- Cluster 4 (Olive): Contains more general terms associated with nature-inspired and swarm intelligence algorithms, including nodes such as nature-inspired algorithm and swarm intelligence. This group encompasses broader theoretical notions, and its connectivity with other clusters reflects the conceptual foundations underlying different research lines. Its low density and limited number of nodes reinforce its role as a theoretical support rather than an operational or practical category.

The distribution of terms within the network suggests the existence of interrelation patterns among different optimization approaches, reflecting the conceptual structure of the field.

**Figure 5:**
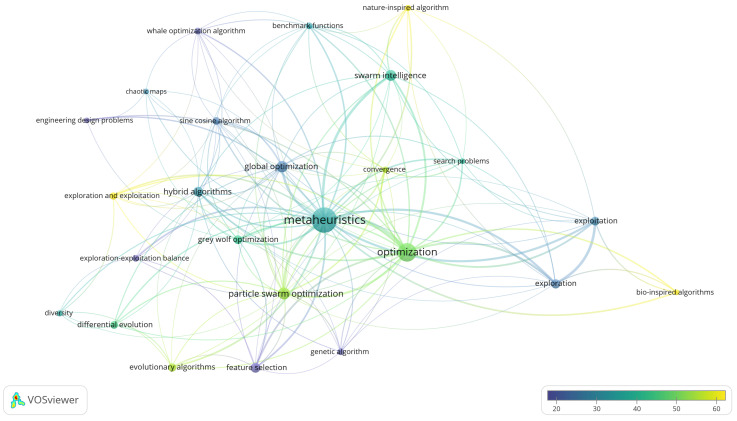
Overlay Visualization: Co-occurrence Network with Average Citations.

As a complement to the structural keyword co-occurrence analysis, an overlay visualization (Figure 5) was generated based on the previously constructed semantic network. This visualization, composed of 25 nodes distributed across four thematic clusters, retains the original node colors while adding an overlay color gradient reflecting the average citations received by documents containing each term. The graphical representation allows for an assessment of the relative bibliometric impact of each term, with the color scale ranging from blue (lower impact) to yellow (higher impact).

The results reveal that:

- Highest average citation values were observed for the terms exploration and exploitation, bio-inspired algorithms, and nature-inspired algorithms, represented in yellow tones. These nodes are primarily located at the periphery of the network, indicating that while their usage frequency is lower, they have had a significant impact in the publications where they appear.
- Medium-high impact terms, depicted in light green, include evolutionary algorithms, particle swarm optimization, optimization, and convergence. These nodes are positioned more centrally within the network, suggesting both a high frequency of occurrence and a moderately elevated citation impact.
- Terms visualized in dark green comprise metaheuristics, grey wolf optimization, hybrid algorithms, swarm intelligence, benchmark functions, differential evolution, diversity, and search problems. These exhibit relatively high occurrence frequency but are associated with lower average citations compared to the aforementioned groups, indicating medium citation impact.
- Lowest average citation terms, represented in blue tones, include exploration, exploitation, feature selection, exploration–exploitation balance, engineering design problems, chaotic maps, and sine cosine algorithm. These predominantly occupy peripheral zones of the network and demonstrate lower accumulated academic visibility and impact, reflecting infrequent citation. Nevertheless, their presence in this region highlights their potential as indicators of emerging trends and specialized applications, suggesting growing research areas.

This visualization enables the identification of not only the thematic structure of the literature but also the relative weight of each node within the bibliometric ecosystem of the research field.

### 3.7. Academic Collaboration Network Analysis

**Figure 6:**
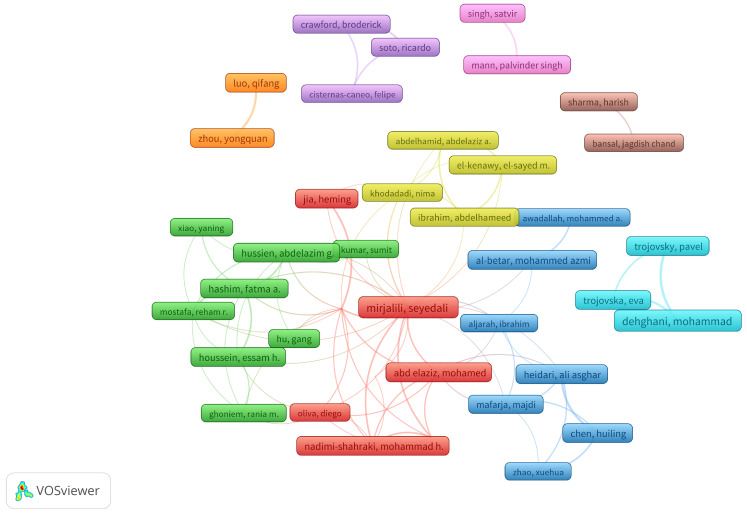
Collaboration Network.

The analysis of academic collaboration networks reveals multiple co-authorship communities, organized into distinct color-coded clusters. The visualization (Figure 6) comprises 36 authors in total, each represented by a node, with connecting links indicating scientific publication collaborations.

The red cluster stands out as the central core of the network, with Seyedali Mirjalili as the most connected node, establishing links with various researchers, including Abd Elaziz, Mohamed, Oliva, Diego, Nadimi-Shahraki, Mohammad H., and Jia, Heming. This group is densely interconnected, evidencing a consolidated community with a high collaboration frequency.

The blue cluster groups a community of researchers with a cohesive collaboration structure. Within this group, the authors Zhao, Xuehua, Chen, Huiling, Mafarja, Majdi, Heidari, Ali Asghar, Aljarah, Ibrahim, Al-Betar, Mohammed Azmi, and Awadallah, Mohammed A. establish recurrent co-authorship links. The connectivity within this cluster indicates the existence of an active collaboration network, focused on specific contributions within the field of study.

The green cluster exhibits a high density of connections among its members, with Abdelazim G. Hussien, Fatma A. Hashim, and Reham R. Mostafa as central authors. The interrelation between these researchers suggests an established community in the production of joint studies.

The yellow cluster, although smaller in size compared to the previous ones, is connected to the main network, indicating its integration into the general collaborative structure. In this group, the authors Khodadadi, Nima, Ibrahim, Abdelhameed, El-Kenawy, El-Sayed M., and Abdelhamid, Abdelaziz A. maintain direct links with researchers from the central core, showing participation in interdisciplinary studies or contribution to cross-cutting research lines within the field.

Additionally, smaller and more peripheral clusters are identified, such as the orange one, formed by Luo, Qifang and Zhou, Yongquan, or the purple cluster, with researchers like Crawford, Broderick, Soto, Ricardo, and Cisternas-Caneo, Felipe. These groups show a lower link density and are not connected to the main network, which may indicate a specialization in more specific research lines or less interaction with the predominant communities in the study area. In particular, the purple cluster has focused on topics such as the binarization of metaheuristics, designed to adapt optimization algorithms to discrete search spaces [38], and the hybridization of metaheuristics with machine learning techniques, aiming to enhance the balance between exploration and exploitation [39]. This reflects a specialized methodological orientation within the field of optimization.

### 3.8. Scientific Contribution by Country

**Figure 7:**
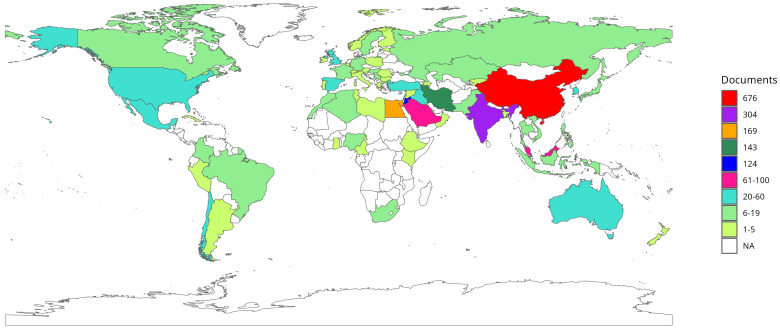
Countries’s Scientific Production.

The cartographic visualization of scientific production by country (Figure 7), based on the institutional affiliations of the authors, displays the geographical distribution of research activity in the analysed subject. In this representation, the same document may be counted in multiple countries if its co-authors are affiliated with institutions located in different nations. Therefore, the total number of publications in this visualization does not correspond to the unique count of documents in the database, but rather to the sum of article appearances across recorded institutional affiliations.

The analysis reveals a predominance of Asia in terms of publication volume. China (red, 676 documents) and India (purple, 304 documents) stand out as the countries with the highest presence in institutional affiliations within the analysed corpus. Egypt (orange, 169 documents), Iran (dark green, 143 documents), and Jordan (blue, 124 documents) complete the group of nations with the highest number of documents counted based on their researchers’ affiliations.

At an intermediate productivity level, Saudi Arabia (deep pink, 96 documents) and Malaysia (deep pink, 95 documents) maintain a notable presence. A third group, represented in turquoise, includes countries with institutional affiliations ranging between 20 and 60 documents, among them Australia (58), the United States (48), Mexico (36), Turkey (35), Iraq (34), South Korea (31), Chile (26), the Czech Republic (26), Spain (24), and the United Kingdom (23).

At an even lower range, countries with 6 to 19 documents are represented in light green, while those with 1 to 5 documents appear in light olive green. Finally, territories with no documents linked to institutional affiliations in the analysed database are depicted in white, highlighting the absence of scientific output across vast regions of Africa and Central America.

## 4. Discussions

The bibliometric analysis reveals that scientific output related to the balance between exploration and exploitation in metaheuristics has experienced exponential growth during the 2005–2024 period, with a particularly notable surge in recent years. The sustained increase in publication volume, along with a marked acceleration starting in 2018, reflects the growing interest and consolidation of this research area. This trend indicates a heightened presence of the topic on the scientific agenda, likely driven by the need to optimize algorithms for solving complex problems in the field of computational intelligence. As of 2024, over 800 publications have been recorded, suggesting that a critical mass of knowledge production has been reached—fostering both the expansion and increasing methodological sophistication of the field. This continuous growth highlights the influence of the exploration–exploitation balance as a key factor in metaheuristic performance, underscoring its relevance in the design of more efficient and adaptive algorithms.

The application of Bradford’s Law highlights an uneven distribution of the scientific literature on this topic. A small number of highly specialized journals account for more than one-third of the total publications. This finding points to the existence of core journals that serve as primary dissemination channels within this research domain. These sources not only concentrate a significant portion of academic activity but also play a central role in shaping and consolidating knowledge on the subject [22]. In contrast, the intermediate and peripheral zones, which encompass the remaining publications, reflect an emerging thematic diversification, with contributions appearing in journals of interdisciplinary scope. Peripheral sources indicate the dissemination of knowledge in outlets that rarely feature work on this topic [22,40]. This dispersion suggests that interest in the exploration–exploitation balance is extending into multiple disciplines, albeit without significant concentration in any single publication venue.

The author productivity and impact analysis highlighted several influential researchers, including Seyedali, Mirjalili, Dehghani, Mohammad, and Abualigah, Laith. Their high publication output, combined with strong h-index and e-index values and consistent citation patterns, confirms their status as key contributors to the development of this research line [15,24,41]. Notably, authors such as Hashim, Fatma A., and Houssein, Essam H., despite having fewer publications, have achieved high citation impact (e-index values of 53 and 48, respectively). This demonstrates that scientific relevance is not solely dependent on the volume of publications, but also on the influence their work has within the academic community.

The incorporation of weighted authorship credit metrics allowed for a more nuanced interpretation of the findings derived from the analysis of gross productivity, revealing substantial differences in the actual distribution of contributions within multi-author publications. Dehghani, Mohammad consistently ranked first across all evaluated metrics, confirming a clear leadership in specialized output on the exploration–exploitation balance. Other authors, such as Trojovsky, Pavel; Houssein, Essam H.; and Trojovska, Eva, also showed a consistent presence among the top ranks, suggesting active and sustained participation in the development of methodological proposals in this domain. The application of multiple credit allocation schemes—with varying sensitivities to author position—strengthened the robustness of the analysis by avoiding biases associated with a single evaluation criterion. These results highlight the need to use more refined metrics to characterize scientific influence, especially in fields where co-authorship is high and authorship order does not always reflect the extent of intellectual contribution [25,26,42,43]. Beyond the influence attributable to co-authorship, the impact of these researchers is also reflected in the citation levels achieved by their most representative publications.

The most cited documents further support this observation, with four out of the ten most highly cited papers authored by Fatma A. H. Her research on adaptive systems and the application of metaheuristic techniques to both engineering problems and standard mathematical benchmarks has established key methodological references for addressing the exploration–exploitation balance. Her 2019 article in Future Generation Computer Systems [10], with 92 local citations in the analyzed database (WoS), stands out as a theoretical cornerstone in the field. In addition, recent works by Braik M. (2022) [29], Fatma A. (2022) [28], and Trojovský (2022) [32] introduce new bio-inspired metaheuristic algorithms, including the White Shark Optimizer (WSO), Honey Badger Algorithm (HBA), and Pelican Optimization Algorithm (POA), respectively. These algorithms aim to improve performance in solving complex optimization problems through strategies that promote an effective balance between exploration and exploitation during the search process.

The keyword co-occurrence analysis revealed a clearly segmented thematic structure, highlighting both well-established lines of research and emerging areas. In this context, the term metaheuristics occupies a central position in the thematic map, reaffirming its role as the dominant methodological core and a key organizing concept in the specialized literature. In contrast, peripheral nodes associated with concepts such as feature selection, diversity, chaotic maps, and engineering design problems indicate the progressive incorporation of more complex and multidimensional approaches. Although these connections appear less frequently, they suggest that the methodologies and theories in this field are being adapted and expanded, enabling the emergence of new research directions that broaden the boundaries of knowledge and drive the field’s evolution [44,45].

The overlay visualization, based on average citations per keyword, offered deeper insight by showing that many of these emerging concepts, despite their lower frequency, exert a significant influence—highlighting their growing prominence in recent literature [44,45,46]. Notably, the term exploration and exploitation stood out due to its high citation count, reinforcing the sustained interest of the scientific community in this conceptual axis and underlining its relevance for the design and evaluation of advanced metaheuristic algorithms. These findings suggest a thematic evolution in which the refinement of the exploration–exploitation balance will become increasingly important, both at the theoretical level and in multidomain applications.

The academic collaboration network analysis revealed a hierarchical structure composed of densely interconnected co-authorship communities, organized into clusters with varying levels of centrality. A core group led by authors with strong institutional connectivity was identified, whose work has been instrumental in consolidating active research communities focused on the exploration–exploitation balance. This pattern of collaboration fosters collective knowledge generation, promotes knowledge transfer, and enhances the global impact of scientific production [46]. However, the visualization also revealed the existence of peripheral clusters with limited interconnection, indicating research lines that are more applied or focused on specific contexts [47,48]. This partial fragmentation presents opportunities to promote interdisciplinary collaborations that integrate both theoretical and practical perspectives, thereby contributing to greater field cohesion and the development of more robust and context-aware methodological approaches.

Finally, the analysis of scientific contributions by country revealed a marked concentration of research activity in a small number of nations. The dominance of China and India, alongside the significant participation of countries in the Middle East and Asia, underscores a strong regional influence in knowledge production. This geographic concentration shapes the dynamics of international collaboration and methodological diversity by reinforcing the consolidation of regionally centered research networks. These patterns have important implications for the future development of exploration–exploitation studies in metaheuristics. They reveal both consolidated strengths and ongoing challenges—particularly the need to foster more global collaboration networks and integrative methodological approaches that can advance the field.

Despite the methodological rigor of this bibliometric analysis, it is important to acknowledge certain inherent limitations. First, the exclusive reliance on the Web of Science database may have constrained the scope of the analyzed corpus by excluding relevant publications indexed in other platforms such as Scopus, IEEE Xplore, or Google Scholar. Additionally, citation counts—while useful as impact indicators—may be influenced by specific dynamics of the academic ecosystem, such as the presence of self-citations or the relatively low citation volume typically associated with recent publications. Another limitation concerns the inclusion criteria applied in the network analysis, such as the use of minimum frequency thresholds for keywords. Although these thresholds enhance statistical robustness, they may also exclude emerging terms of growing relevance. On the other hand, it is recognized that the quantitative approach adopted limits the in-depth analysis of the methodological quality and substantive contributions of the studies considered [49,50]. Despite the application of standardization and terminological filtering processes, the keywords used do not always accurately reflect the thematic focus or the real contribution of each paper [51,52], which may limit the representativeness of the cooccurrence maps and condition the interpretation of the semantic clusters. Likewise, although bibliometric data are objective, their analysis requires interpretative judgments that introduce a certain degree of subjectivity [50,53,54,55]. It is recommended that this type of study be complemented with systematic reviews or qualitative approaches that allow dimensions not captured by bibliometric analysis to be addressed [56]. In line with this orientation, future research will carry out a systematic review that will allow a qualitative and critical examination of the corpus analyzed, in order to complement and contrast the findings obtained by means of quantitative techniques. Nonetheless, despite these limitations, the findings presented here offer a solid foundation for understanding the evolution of this research line and for guiding future work toward more integrative, dynamic, and context-sensitive approaches.

## 5. Conclusions

It has been evidenced that the scientific production on the exploration–exploitation balance in metaheuristics has experienced a sustained and exponential growth between 2005 and 2024, with a remarkable acceleration from 2018 onwards. This increase reflects a growing interest on the part of the research community in improving the performance of algorithms by dynamically adjusting between diversification and intensification throughout the search process. The results obtained allow us to identify thematic gaps, emerging areas and patterns of scientific collaboration on the balance between exploration and exploitation in bio-inspired algorithms. This information can be useful to guide future research, define systematic review agendas and support editorial or evaluative processes in the field of optimization. Regarding the distribution of publications, it has been found that a small number of specialized sources account for more than one third of the total, in accordance with Bradford’s Law. The intermediate and peripheral zones, on the other hand, show a thematic diversification towards interdisciplinary journals. This configuration suggests the existence of key channels for the dissemination of knowledge and, at the same time, an incipient deployment of the subject matter in related fields. At the thematic level, it has been observed that the terms “exploration and exploitation” have a high citation impact despite their lower frequency as keywords, which underlines their emerging relevance in the design and evaluation of advanced algorithms. Likewise, consolidated lines of research (e.g., particle swarm optimization, convergence) have been identified along with emerging areas in expansion (such as feature selection, exploration–exploitation balance, chaotic maps), which is evidence of a constantly evolving field of study. In this line, future research is expected to capitalize on the opportunities detected in this bibliometric analysis through the design and validation of hybrid and adaptive strategies, aimed at optimizing the performance of metaheuristics in the face of highly complex problems. In terms of academic collaboration, the observed structure is characterized by densely interconnected communities led by highly central researchers, as well as by peripheral clusters with a lower degree of integration. This partial fragmentation highlights the opportunity to foster interdisciplinary synergies and to strengthen the links between consolidated and emerging groups. Finally, at the geographic level, it has been found that scientific production is strongly concentrated in Asia, with China and India leading the way. This pattern poses the challenge of expanding international collaboration networks and diversifying methodological perspectives on the subject.

## Figures and Tables

**Table 1:** Scientific production.

Description	Results
Document information	
Timespan	2005:2024
Number of journals	293
Number of documents	861
Author’s Keywords	2267
Document Average Age	3.94
Average citations per document	32.08
Author information	
Number of authors	2392
Number of single-authored documents	40
Average number of co-authors per document	3.81

**Table 2:** Impact of scientific production.

Author	Docs	Cites	h-Index	e-Index
Mirjalili Seyedali	27	2966	21	50
Dehghani Mohammad	22	1331	18	32
Abualigah Laith	21	1092	14	30
Chen Huiling	13	617	10	23
Hashim Fatma A.	13	2952	11	53
Jia Heming	13	526	10	21
Trojovsky Pavel	13	1121	11	32
Heidari Ali Asghar	12	560	10	21
Houssein Essam H.	12	2385	10	48
Abd Elaziz Mohamed	11	882	9	28
Al-Betar Mohammed Azmi	11	920	9	29
Zhou Yongquan	11	219	7	13
Hussien Abdelazim G.	9	1026	8	31
Luo Qifang	9	185	7	12
Nadimi-Shahraki Mohammad H.	9	604	9	23
Trojovska Eva	9	465	9	20
Crawford Broderick	8	43	4	5
Mafarja Majdi	8	374	8	18
Soto Ricardo	8	43	4	5
Wang Ling	8	559	6	23

**Table 3:** Author Credit Allocation Using Four Weighting Schemes in the Exploration–Exploitation Domain.

Author	Fractional	Harmonic	Geometric	Arithmetic
Dehghani Mohammad	7.54–(1)	7.69–(1)	7.94–(1)	7.52–(1)
Trojovsky Pavel	5.12–(3)	4.62–(2)	4.44–(4)	4.63–(2)
Houssein Essam H.	3.03–(8)	4.18–(3)	4.55–(2)	3.97–(3)
Trojovska Eva	3.12–(6)	3.86–(5)	3.95–(6)	3.93–(4)
Jia Heming	3.33–(5)	3.76–(7)	4.05–(5)	3.67–(6)
Hashim Fatma A.	3.00–(9)	4.14–(4)	4.45–(3)	3.53–(8)
Mirjalili Seyedali	5.56–(2)	3.30–(8)	2.39–(16)	3.61–(7)
Abualigah Laith	3.78–(4)	3.04–(10)	2.69–(13)	3.27–(9)
Al-Betar Mohammed Azmi	3.05–(7)	2.92–(11)	2.92–(11)	3.22–(10)
Nadimi-Shahraki Mohammad H.	2.18–(17)	3.21–(9)	3.70–(8)	2.90–(11)
Abd Elaziz Mohamed	2.55–(13)	2.79–(12)	2.76–(12)	2.53–(14)
Zhou Yongquan	2.90–(10)	2.65–(16)	2.59–(15)	2.71–(12)

**Table 4:** Most Local Cited Documents.

Rank	First Author	Journal	Reference	Year	Total Citations
1	Fatma A.H	Future Generation Computer Systems	[10]	2019	92
2	Fatma A.H	Applied Intelligence	[27]	2021	67
3	Fatma A.H	Mathematics and Computers in Simulation	[28]	2022	58
4	Malik B.	Knowledge-Based Systems	[29]	2022	49
5	Mohamed A.E	Expert Systems with Applications	[30]	2017	39
6	Fatma A.H	Knowledge-Based Systems	[31]	2022	38
7	Ayyarao T.S.L.V	IEEE Access	[6]	2022	27
8	Morales-Castañeda B	Swarm and Evolutionary Computation	[1]	2020	25
9	Trojovský P	Sensors	[32]	2022	24
10	Aydilek I.B	Applied Soft Computing	[33]	2018	20
